# Knowledge and Practice of Pharmacists toward Antimicrobial Stewardship in Pakistan

**DOI:** 10.3390/pharmacy6040116

**Published:** 2018-10-23

**Authors:** Inayat Ur Rehman, Malik Muhammad Asad, Allah Bukhsh, Zahid Ali, Humera Ata, Juman Abdulelah Dujaili, Ali Qais Blebil, Tahir Mehmood Khan

**Affiliations:** 1 School of Pharmacy, Monash University, Jalan Lagoon Selatan, Bandar Sunway, 47500 Selangor, Malaysia; Allah.Bukhsh@monash.edu (A.B.); jumandujaili@yahoo.com (J.A.D.); aliblebil@yahoo.com (A.Q.B.); 2 Department of Pharmacy, Abdul Wali Khan University, Mardan 23200, Pakistan; 3 Department of Pharmaceutical services, Jinnah Postgraduate Medical Center, Karachi 75510, Pakistan; malickasad@gmail.com; 4 Institute of Pharmaceutical Sciences, University of Veterinary and Animal Sciences, Lahore 54000, Pakistan; 5 Department of Pharmacy, University of Peshawar, Peshawar 25210, Pakistan; zahidd.alii@gmail.com; 6 Maternal, Newborn, and Child Health (MNCH) Project Coordinator at Frontier Primary Health Care, Mardan 23200, Pakistan; niazihumera@gmail.com

**Keywords:** practice, knowledge, antimicrobial stewardship, pharmacist, Pakistan

## Abstract

**Background:** The irrational use, “over the counter supply”, and unregulated supply chains of antimicrobials are contributing toward antimicrobial resistance. Antimicrobial stewardship programs regulate antimicrobials usage to prevent resistance and reduce health care burden. **Objective:** To assess the knowledge and practice of pharmacists’ working in various healthcare settings toward antimicrobial stewardship in Pakistan. **Method:** A cross-sectional study was conducted among pharmacists working in different sectors between March to June 2017. **Results:** A total of 181 pharmacists participated, of whom (*n* = 145, 80.1%) were males. The majority of participants were in the 20–30 age group (*n* = 147, 81.2%) and hold Doctor of Pharmacy degrees. More than 80% of pharmacists agreed that “antimicrobial stewardship is essential to improve patient care”; while (*n* = 159, 87.8%) pharmacists agreed that “pharmacist should be trained on the use of antimicrobial”. Close to 90% of pharmacists agreed that “adequate training should be provided to pharmacists on antimicrobial use”. Regarding the practice of antimicrobial stewardship, (*n* = 72, 39.8%) pharmacists often/always “make efforts to prevent or reduce the transmission of infections within the community”; (*n* = 58, 32%) pharmacists never “dispense antimicrobials without a prescription”; and (*n* = 60, 32%) pharmacist often/always “communicate with prescribers if unsure about the appropriateness of an antibiotic prescription”. **Conclusions**: Increased antimicrobial stewardship efforts can both optimize the treatment of infections and reduce adverse events associated with antibiotic use. Pharmacists in Pakistan have good knowledge and adopt positive practices toward antimicrobial stewardship. Pharmacist and other health care professionals should collaborate within multi-disciplinary teams to reduce the problem of antimicrobial resistance and improve the quality of life of patients.

## 1. Background

The elevated incidence of infections due to “multi-drug” resistant bacteria is among the leading causes of mortality worldwide [1]. Where both developed as well as developing countries are facing a crisis because of this. In the United States (US), the estimated number of people affected by infections due to antimicrobial-resistant bacteria totalled up to 2 million per year, with resulting death in about 23,000 people with various types of infections [2]. In European countries, antimicrobial resistance is also on the rise and considered to be responsible for about 25,000 deaths annually [3], while globally it has resulted in 700,000 deaths per year [3]. Indeed, it is projected that by 2050, deaths due to antimicrobial resistance will rise to such a level that it will surpass deaths due to cancer [3]. The World Health Organization (WHO) reports that antimicrobial-resistant microorganisms reduce the effectiveness of drugs, prolonging the duration of infection and ultimately hospitalization [4]. The issue of antimicrobial resistance is worse in low and middle-income countries [LMIC], as the incidence of infectious diseases is high compared to high-income countries [5]. In low and middle-income countries, the mortality rates due to antimicrobial-resistant bacteria are under-reported [5], however, available data in India, Nigeria, Pakistan, and the Democratic Republic of Congo indicate that a huge number of neonatal deaths resulted from drug-resistant sepsis [6].

The safety of drugs prescribed gained importance due to adverse drug reactions caused by commonly used medications [7]. The use of antimicrobials always carries a risk toward both individual patients and public health, where the misuse and overuse of antimicrobials are linked to severe problems toward patient safety resulting from antimicrobial resistance [8,9,10,11], multiple drug resistance [12], elevated cost for health services [13,14], and side effects [11,14]. The major reasons for antimicrobial resistance in low and middle-income countries are a misuse of antimicrobials because they are easily available without a prescription and unregulated supply chains [15,16]. Findings of previous studies also revealed that almost 20–50% of antimicrobials consumed by patients are inappropriate [17]. A pharmacist dispensing antimicrobials without a prescription is 83–100% of the time unaware of a patient’s allergies status [18,19].

Antimicrobial stewardship (AMS) is a term jointly used for various quality improvement activities related to antimicrobials with a major focus on the appropriate and rational use of antimicrobials utilized for the prevention and treatment of infectious diseases. Due to the global increase in the number of cases and prevalence of multidrug-resistant bacteria, the interest in the implementation of AMS has gained importance to address the situation and promote appropriate antimicrobial use with the aim of avoiding more cases of antimicrobial resistance [20]. In the majority of European countries, AMS was implemented in order to reduce the abuse and misuse of antimicrobials, and the associated risk of resistance and adverse events [21]. Furthermore, the AMS program is dedicated to regulate antimicrobials usage in order to prevent resistance to antimicrobials and also aimed to reduce the burden on the health care system [21]. Pharmacists play a vital role in combating and preventing infectious diseases, and their role in AMS programmes have flourished significantly over the past few decades [22,23].

In Pakistan, the existence of more than 600,000 non-registered medical practitioners, poor prescriptions, self-medication, substandard and spurious availability of medicines, easy availability of over the counter antimicrobials by retail pharmacy shops, and false advertisements of antibiotics are major threats to the future of antibiotics and antimicrobial resistance [24]. Various studies have reported higher prescription rates of antimicrobials in Pakistan i.e., 51.5% [25], 52.4% [25], and 57.2% [26]. In Pakistan, a national antimicrobial resistance surveillance system was implemented on recommendation by the WHO, as per recommendation in November 2015 [27], Medical Microbiology and Infectious Diseases Society of Pakistan initiated the AMS program in Pakistan among public and private institutions [28]; and a lot of research has been done to assess the knowledge and perception of medical health professionals toward antimicrobial use and AMS. Despite the presence of large well-established public hospitals across Pakistan, no AMS or stewardship teams exist. A survey of 11 major private and public hospitals in large cities showed that only a few have functioning stewardship activities [29]. A shortage of human resources (infectious diseases physicians, microbiologists, clinical pharmacists, properly trained infection control and prevention nurses, laboratory and microbiology technologists/technicians, funding etc.) was identified in all hospitals [29]. However, very limited or no work has been done to assess the knowledge and practice of pharmacists toward AMS. As such, this study aimed to assess the knowledge and practice of pharmacists in various healthcare settings toward AMS.

## 2. Methods

### 2.1. Study Design and Settings

A descriptive, cross-sectional study was conducted among pharmacists working in different sectors (government and private hospital), community pharmacists in an urban setting only and with different job descriptions like hospital and community pharmacy in Pakistan, to assess the knowledge and practice of pharmacists toward AMS. The duration of the study was for a 4 month-period, ranging from March to June 2017.

### 2.2. Data Source

Demographics of the pharmacists and their background were kept in mind throughout the research/activity process, which was of great importance especially when dealing with antimicrobials. Only pharmacists dealing with antimicrobials in the hospital, retail/community settings were included, while pharmacists who were not willing to participate were excluded for this study. Verbal consent was obtained from all respondents to participate in this study.

### 2.3. Ethics Approval

The study was reviewed and approved by the Jinnah Postgraduate Medical Center Karachi, Pakistan.

### 2.4. Study Questionnaire

A validated questionnaire by Khan et al. [30] was used, which consisted of 26 questions divided into three sections. The first section was related to participants’ demographic information; the second section contained eight questions to assess participants’ knowledge of AMS, while the third section consisted of 11 questions related to participants’ practice toward AMS. For section two, responses were collected on a Likert scale (strongly disagree, disagree, neutral, agree, and strongly agree), while for section three, responses related to frequency were collected on a Likert scale using never, rare, occasionally, often, and always as responses.

### 2.5. Data Analysis

Data were analyzed using SPSS version 22.0 for Windows (Statistical Package for Social Sciences, Chicago, IL, USA). Descriptive analysis was used to express data as frequencies and percentages. A non-parametric test (Kruskal-Wallis test) was used and items were ranked base on the important relative index (RII) values shown in Equation (1) [31]. The important relative index (RII) was used to assess the knowledge and practice of pharmacists toward AMS in Pakistan, where the items having a relative index (RII) value closest to one was ranked as the main factor in the knowledge and practice of pharmacists toward AMS in Pakistan. A *p*-value of <0.05 was considered statistically significant [31].

(1) $$\mathrm{RII}=\frac{\sum W}{\mathrm{AXN}}\left(0\leq \mathrm{RII}\leq 1\right)$$

In Equation (1), W is the weight given to each item by the respondents on a scale from 1–5, (where ‘1’ = strongly disagree and ‘5’ = strongly agree) for the knowledge of pharmacists toward AMS, while 1 = never, 2 = rare, 3 = Occasionally, 4 = often, and 5 = Always, for the practice of pharmacists toward AMS; **A** is the highest weight (i.e., 5 in this case); and **N** is the total number of respondents.

## 3. Results

### 3.1. Demographics

A total of 250 pharmacists were approached, only 181 filled the questionnaire and the response rate was 72.4%. The majority of pharmacists who participated in this study were males (*n* = 145, 80.1%) and in the 20–30 year age group (*n* = 147, 81.2%). The majority held Doctor of Pharmacy degrees (Pharm.D) (*n* = 132, 72.9%) and work in the hospital setting *n* = 93 (51.4%), followed by the community pharmacy setting (*n* = 67, 37.0%) and clinical pharmacy setting (*n* = 15, 8.3%). Most of the respondents had 2–3 years of job experience (*n* = 66, 36.5%), followed by <1 year (*n* = 57, 31.5%) and >5 years (*n* = 31, 17.1%) (details are shown in Table 1).

### 3.2. Knowledge of Pharmacists of Antimicrobial Stewardship

Overall, (*n* = 152, 83.9%) pharmacists agreed that “*antimicrobial stewardship is essential to improve patient care*” (*p* = 0.430), while (*n* = 159, 87.8%) pharmacists agreed that “*pharmacist should be trained on the use of antimicrobial*” (*p* = 0.459). Approximately 85% of pharmacists agreed that for “*enhancing the understanding of antimicrobial stewardship pharmacist should be given exposure to relevant workshops, conferences, and workshops activity*” (*p* = 0.701), and that “*antimicrobial stewardship programs reduce the problem of antimicrobial resistance*” (*p* = 0.897, as shown in Table 2. None of the statements regarding the knowledge of pharmacists of AMS were statistically significant. Variation in responses made it impossible to rank the main statements regarding the knowledge of AMS, therefore, the important relative index (RII) was used to estimate the relative importance of the identified statements. The RII analysis revealed that top five statements with regard to knowledge of pharmacist knowledge toward AMS were “*training should be imparted to pharmacists on use of antimicrobial*” RII = 0.87; “*for understanding of antimicrobial stewardship pharmacist needed to attend relevant conferences, workshops for better understanding*” RII = 0.86, “*pharmacists have a responsibility to take prominent role in antimicrobial stewardship and infection control programs in health system*” RII = 0.86, “*antimicrobial stewardship programs reduce problem of antimicrobial resistance*” RII = 0.83, “*antimicrobial stewardship programs improve patient care*” RII = 0.82, and “*antimicrobial stewardship should be incorporated at pharmacy level*” RII = 0.81. However, the statements “*individual efforts at antimicrobial stewardship has minimal impact antimicrobial resistance problem*” RII = 0.67 and “*I think that the prescribing physicians are the only professionals who need to understand antimicrobial stewardship*” RII = 0.34, was ranked the lowest (details are shown in Table 2).

### 3.3. Practice of Pharmacists toward Antimicrobial Stewardship

Close to 40% of the pharmacists “make efforts to prevent or reduce the transmission of infections within the community” (*p* = 0.045); (*n* = 58, 32%) never “dispense antimicrobials without a prescription” (*p* = 0.006), (*n* = 60, 32%) “communicate with prescribers if I am unsure about the appropriateness of an antibiotic prescription” (*p* = 0.001), and (*n* = 52, 28.7%) “ask the patients about their knowledge of prescribed antimicrobial and its usage” (*p* = 0.151), as shown in Table 3. Variation in responses made it impossible to rank the main statements regarding the practice of pharmacists toward AMS, therefore, the important relative index (RII) was used to estimate the relative importance of the identified statements. The RII analysis revealed top six ranked practices by pharmacists in Pakistan toward AMS “make efforts to prevent or reduce the transmission of infections within the community”, RII = 0.77; “educate patients on the use of antimicrobials, and resistance-related issues”, RII = 0.77; “dispense antimicrobial on prescription with complete clinical information”, RII = 0.72; “communicate with prescribers if I am unsure about the appropriateness of an antibiotic prescription”, RII = 0.71; “sought additional clinical information e.g., drug interaction, ADRs, allergy, etc.”, RII = 0.69; and “ask the patients about their knowledge of prescribed antimicrobial and its usage”, RII = 0.69 (Table 3).

## 4. Discussion

This is the first study to explore the knowledge and practice of pharmacists working in the hospital, clinical and community setting toward AMS in Pakistan. Overall, the current sample was an appropriate and accurate representation of pharmacists working in the health care setting directly involved in dispensing and counselling patients about antimicrobials. In the current scenario, due to resistance toward antimicrobials worldwide, establishment and practical utilization of AMS programs have gained a lot of importance to conserve the efficacy of antimicrobials.

Patient safety is an integral component of AMS, and as such, the AMS should be viewed as a safety initiative for both patients and public health [32]. Worldwide, AMS programs have been implemented to optimize antimicrobial therapy in order to improve patient safety and the quality of care [33]. Previous studies have reported the suboptimal and unnecessary use of antibiotics of about 25% to 50% in hospitals [34,35,36,37]. Misuse of antimicrobials can lead to antibiotic resistance, which adversely impacts both morbidity and mortality, and increases the length of stay in the hospital and cost [10,38,39,40,41]. An effective AMS program can reduce the improper use of antimicrobials, duration of hospitalization, resistant infection rate, and cost [42]. Pharmacists being active members of the health care system have leading roles in AMS and infection control programs to protect patients from developing resistance against antimicrobials and to establish a good health care system.

Pakistan being a developing country has witnessed many cases of antimicrobial resistance due to irrational use. The Medical Microbiology and Infectious Diseases Society of Pakistan initiated an AMS program [43], however, at the government level, a national action plan for AMS and infection control is needed in Pakistan [44]. In our study, almost all pharmacists were aware of AMS programs, and have a good knowledge and practice toward AMS in Pakistan; Similar findings were also reported in a study done in Punjab (province of Pakistan) among pharmacist [45]. Approximately 80% of pharmacists were in favour and agreed that AMS programs improve patient care, which is aligned with the findings by Erku DA et al., where 86.3% of pharmacists were in favour of this. Research in other parts of the world also supports our findings that pharmacists and other health care professionals were in favour of an AMS program [46]. Similarly, AMS has been implemented in the majority of European countries in order to reduce the abuse and misuse of antimicrobials, the associated risk of resistance and adverse events [21] expenditure, the prevalence of resistant pathogens [47,48], and improve clinical outcomes [47,49]. In our study, 87.8% pharmacist agreed that suitable training should be offered to pharmacists on the appropriate use of antimicrobials, similar with the findings of Crader M et al. that for all pharmacists in the hospital setting, antimicrobial steward programs should be initiated, and antibiotic competency and training should be offered [45,50]. Similarly, Dellit TH et al. stated that pharmacists should have infectious disease training to work in hospitals in order to optimize antibiotic stewardship [51]. Our study revealed that 85.6% of pharmacist agreed that relevant conferences, workshops, and other educational activity are required to be attended by pharmacists to enhance their understanding of AMS, while 84.5% agreed that pharmacists have a major role in AMS to control infection. Our findings are similar to another study in which 90.5% of pharmacists were in favour of relevant conferences, workshops and seminars [52], and also highlighted the prominent role of pharmacists in AMS programs [52,53]. 

Regarding the practice of pharmacists with regard to antimicrobial dispensing, our study found that 30.4% and 28.2% of pharmacists dispensed antimicrobials only when prescribed quite often and always, respectively. Similarly, 32.0% and 28.2% of pharmacists responded that they never and rarely dispense antimicrobials without a prescription respectively. Our results are similar to the finding of Khan et al. in which 36.7% of pharmacists never, while 47.3% rarely dispensed antimicrobial without a prescription [30]; however, the findings of Erku et al. revealed that 59.9% of pharmacists often/always dispense antimicrobials without a valid prescription [52]. While another study in Pakistan showed that community pharmacists dispensed antibiotics without a prescription [45]. In our study, 65.7% of pharmacists never, while 14.9% of pharmacists rarely dispense antimicrobials if patients request for longer durations than that prescribed by their physicians; while in the study by Erku et al., about 39.6% often and 19.8% occasionally [52]; and in the Khan et al. study, 39.4% pharmacists rarely, and 19.6% occasionally [30] dispense antimicrobials for more than the prescribed period. From our findings, 26.5% of pharmacists responded that they quite often, and 40.9% responded that they always provide education to patients on antimicrobials use and resistance related issues. This is different from the findings by Khan et al. in which 61.2% of pharmacists often, and 10.1% always educate patients on the use of antimicrobials and resistance [30]. Lastly, in our study, 27.1% of pharmacists responded that they often, and 39.8% always make efforts for infection transmission within the community, which are different from that found in the study by Eruk et al. in which 36.6% of pharmacists often and 28.8% occasionally make efforts to decrease the spread of infection in the community [52].

In Pakistan, the community pharmacy settings have no well-established AMS programs, but fortunately, the community pharmacists are well aware of their roles in AMS programs. A positive understanding of AMS must be utilized for the betterment of patients [45]. Overall, our study revealed that pharmacists demonstrated good knowledge and positive practice toward AMS but as discussed, a national AMD program should be implemented in Pakistan. The findings of this research will encourage the pharmacists working in the different sectors of health care to raise awareness about antimicrobial resistance and advocate at a different level about the implementation of AMS in government as well as private hospital levels to ensure safety and low cost to the patients and society. Similarly, in each tertiary health care setting, an indigenous AMS program should be implemented to address antimicrobial resistance. Pharmacists and other health care professionals should work together in multidisciplinary teams in AMS programs to reduce resistance toward antimicrobials and to reduce the irrational and inappropriate use of antimicrobials. This is to ensure patients have a good quality of life, decrease cost burden on the patient as well as government, and reduce hospitalization. 

## 5. Strength and Limitations

The strength of this study is that it is the first such study conducted among pharmacists working in government/private hospitals, community pharmacy settings in Pakistan, and highlighted key points to implement AMS on a national level and in individual hospitals and community pharmacies. The findings of this study will assist in the design of larger-scale studies to assess the knowledge and practice of pharmacists toward AMS, with the end goal of reducing antimicrobial resistance in Pakistan. A limitation of this project is that limited literature was available on this topic. The sample size was also limited, and participants were approached randomly from a few cities in Pakistan. As such, the findings cannot be generalized to all pharmacists working in hospital, clinical, or community pharmacies.

## 6. Conclusions

Overall, the study revealed that pharmacists in Pakistan have a positive knowledge and practice toward AMS. The implementation of AMS programs is needed in Pakistan and pharmacists should play a vital role in it by improving and updating their knowledge according to current guidelines with continuous training and professional development programs. Pharmacists and other health care professionals should collaborate within multi-disciplinary teams to reduce the risk of antimicrobial resistance, thereby reducing the economic burden, improving patients’ quality of life, and reducing hospitalization due to infections. Increased AMS efforts can both optimize the treatment of infections and reduce adverse events associated with antibiotic use. Moreover, AMS can help pharmacists improve the quality of patient care and improve patient safety through increased infection cure rates, reduced treatment failures, and increased frequency of correct prescribing for therapy and prophylaxis.

## Figures and Tables

**Table 1:** Demographics of respondents (*n* = 181).

Variables	*n* (%)
**Gender**	
Male	145 (80.1%)
Female	36 (19.9%)
**Age**	
20–30 Years	147 (81.2%)
31–40 Years	32 (17.7%)
41–50 Years	2 (1.1%)
**Education**	
Bachelor of Pharmacy	8 (4.4%)
Doctor of Pharmacy	132 (72.9%)
Master of Philosophy	35 (19.3%)
Master of Public Health	1 (0.6%)
Doctor of Philosophy	5 (2.8%)
**Job Status**	
Clinical Pharmacist	15 (8.3%)
Community Pharmacist	67 (37.0%)
Hospital Pharmacist	93 (51.4%)
Another sector **	6 (3.4%)
**Job Experience**	
<1 year	57 (31.5%)
>5 years	31 (17.1%)
2–3 years	66 (36.5%)
4–5 years	27 (14.9%)

** Another sector: Pharmacists working as educators (diabetic educator or as a specific disease-related education).

**Table 2:** Knowledge of participants towards antimicrobial stewardship.

Statements	Strongly Agree	Agree	Neutral	Disagree	Strongly Disagree	RI	Rank	*p*-Value
Antimicrobial stewardship programs improve patient care	72 (39.8%)	80 (44.2%)	12 (6.6%)	6 (3.3%)	11 (6.1%)	0.82	4	0.430
Antimicrobial stewardship should be incorporated at pharmacy level	77 (42.5%)	69 (38.1%)	13 (7.2%)	12 (6.6%)	10 (5.5%)	0.81	5	0.398
Antimicrobial stewardship programs reduce problem of antimicrobial resistance	85 (47.0%)	66 (36.5%)	14 (7.7%)	6 (3.3%)	10 (5.5%)	0.83	3	0.897
Adequate training should be provided to pharmacists on antimicrobial use	112 (61.9%)	47 (26.0%)	5 (2.8%)	6 (3.3%)	11 (6.1%)	0.87	1	0.459
Relevant conferences, workshops and other educational activity are required to be attended by a pharmacist to enhance understanding of antimicrobial stewardship	111 (61.3%)	44 (24.3%)	8 (4.4%)	8 (4.4%)	10 (5.5%)	0.86	2	0.701
Individual efforts at antimicrobial stewardship has minimal impact antimicrobial resistance problem	19 (10.5%)	74 (40.9%)	49 (27.1%)	25 (13.8%)	14 (7.7%)	0.67	6	0.228
I think that the prescribing physicians are the only professionals who need to understand antimicrobial stewardship	15 (8.3%)	29 (16.0%)	20 (11.0%)	57 (31.5%)	60 (33.1%)	0.34	7	0.391
Pharmacists have a responsibility to take prominent role in antimicrobial stewardship and infection control programs in health system	113 (62.4%)	40 (22.1%)	11 (6.1%)	6 (3.3%)	11 (6.1%)	0.86	2	0.410

Kruskal-Wallis test was applied, RI = relative index, Grouping variable job sector.

**Table 3:** Practices of participants towards antimicrobial stewardship.

Statements	Never	Rare	Occasionally	Often	Always	RI	Rank	*p*-Value
I dispense antimicrobial on prescription with complete clinical information	11 (6.1)	26 (14.4)	38 (21.0)	55 (30.4)	51 (28.2)	0.72	2	0.106
I dispense antimicrobials without a prescription	58 (32.0)	51 (28.2)	37 (20.4)	30 (16.6)	5 (2.8)	0.46	8	0.006 *
I dispense antimicrobial for durations more than prescribed by the physician on patient request	119 (65.7)	27 (14.9)	19 (10.5)	9 (5.0)	7 (3.9)	0.33	9	0.478
I screen the antimicrobial prescription in accordance with local guidelines before dispensing	21 (11.6)	45 (24.9)	29 (16.0)	53 (29.3)	33 (18.2)	0.64	6	0.110
I collaborate with other health professionals for infection control and Antimicrobial stewardship	28 (15.5)	32 (17.7)	37 (20.4)	49 (27.1)	35 (19.3)	0.63	7	0.538
I communicate with prescribers if I am unsure about the appropriateness of an antibiotic prescription	17 (9.4)	37 (20.4)	27 (14.9)	42 (23.2)	60 (32.0)	0.71	3	0.001 *
I sought additional clinical information (E.g., drug interaction, ADRs, allergy, etc.)	16 (8.8)	34 (18.8)	34 (18.8)	45 (24.9)	52 (28.7)	0.69	4	0.134
I take part in antimicrobial awareness campaigns to promote the optimal use of antimicrobials	20 (11.0)	33 (18.2)	40 (22.1)	48 (26.5)	40 (22.1)	0.66	5	0.008 *
I educate patients on the use of antimicrobials, and resistance-related issues	9 (5.0)	22 (12.2)	28 (15.5)	48 (26.5)	74 (40.9)	0.77	1	0.179
I make efforts to prevent or reduce the transmission of infections within the community	7 (3.9)	24 (13.3)	29 (16.0)	49 (27.1)	72 (39.8)	0.77	1	0.045 *
I ask the patients about their knowledge of prescribed antimicrobial and its usage	17 (9.4)	31 (17.1)	37 (20.4)	44 (24.3)	52 (28.7)	0.69	4	0.151

Kruskal-Wallis test was applied, RI = relative index, * *p*-value < 0.05 was considered statistically significant. Grouping variable job sector.

## Declarations

Ethics Approval. Ethics approval obtained from Jinnah postgraduate medical centre Pakistan.

## Abbreviations

AMS	antimicrobial stewardship
CDC	Center for disease control and prevention
WHO	World health organization
RII	important relative index
